# Central Nervous System Tuberculoma

**DOI:** 10.4269/ajtmh.20-1495

**Published:** 2021-04-26

**Authors:** Juan Cataño, Jessica Porras

**Affiliations:** 1 Infectious Diseases Section, Internal Medicine Department, University of Antioquia School of Medicine, Medellín, Colombia;; 2 Infectious Diseases Section, CES Clinic, Medellín, Colombia

**Figure 1.**
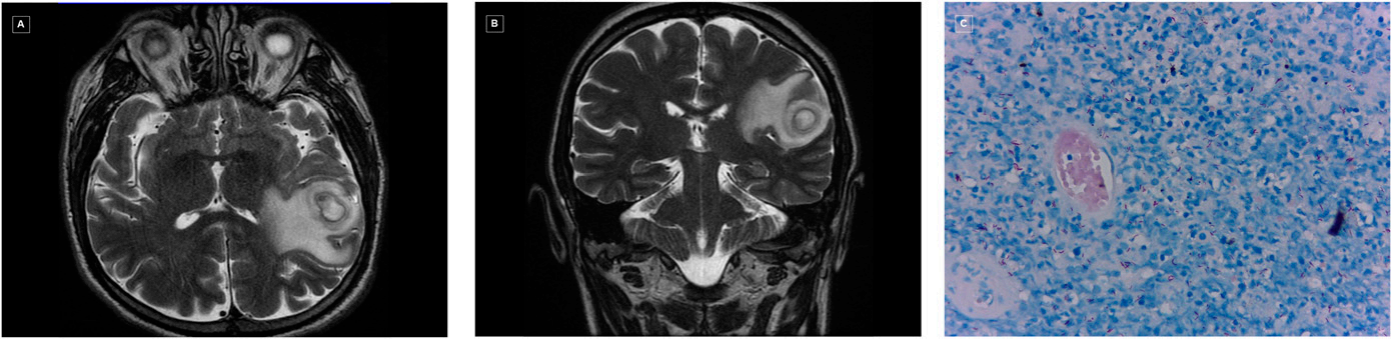
(**A** and **B**) Contrasted magnetic resonance imaging of the head (axial y coronal view), showing a left parietotemporal lesion with ring-enhancing after gadolinium administration, and perilesional vasogenic edema. (**C**) Histologic examination of the brain lesion, showing liquefaction necrosis and abundant acid-fast bacilli on Ziehl-Neelsen stain. This figure appears in color at www.ajtmh.org.

A 54-year-old man from a rural town in Colombia and with well-controlled hypertension and dyslipidemia presented with 3 months of malaise, 10-kg weight loss, and 1 month of headache and progressive dysphagia. He denied fever and other symptoms. On examination, he was alert, oriented, afebrile, blood pressure was 108/69 mm Hg, pulse 72 beats/min, respiratory rate 18/minute, oxygen saturation 97% on room air, weight 52 kg, and body mass index 19. Neurological examination showed right hemiparesis and hyperreflexia. Magnetic resonance imaging of the head showed an oval lesion in the left parietotemporal region, with ring-enhancement after the administration of gadolinium; perilesional vasogenic edema with minimum displacement of the midline was present (Figure 1A and B). Because of suspicion of a central nervous system (CNS) neoplasm, 2 days later craniotomy was performed; histologic examination showed liquefaction necrosis, and acid-fast bacilli were demonstrated on Ziehl-Neelsen staining (Figure 1C). A test for HIV was positive, with CD4 count of 121 cells/mL and HIV viral load of 600,000 copies/mL, with no evidence of pulmonary disease by chest contrasted tomography. *Mycobacterium tuberculosis* was confirmed using molecular methods; isoniazid, ethambutol, rifampin, and pyrazinamide along with pyridoxine were started, and the patient was discharged 1 week later with right hemiparesis and hyperreflexia as sequelae.

Tuberculosis is common in low- and middle-income countries, where CNS involvement accounts for 5% to 10% of all *M. tuberculosis* infections, mainly in children and immunosuppressed patients, particularly those infected with HIV.^1^ Involvement of the CNS, compared with involvement of other systems, represents the most severe form of the disease, conferring the highest rates of morbidity and mortality, even in patients who are adequately treated. Tuberculosis of the brain remains a diagnostic challenge due to nonspecific findings that mimic CNS neoplasms.^2^ Stereotactic biopsy with histopathological analysis can provide a definitive diagnosis but is only recommended when less invasive methods, such as lumbar puncture with molecular analysis of the cerebrospinal fluid, are inconclusive. Standard medical treatment with four drugs can cure the infection, but neurological sequelae can be devastating depending on the brain area involved.^3^
